# Measuring dose-related efficacy of eptinezumab for migraine prevention: post hoc analysis of PROMISE-1 and PROMISE-2

**DOI:** 10.1186/s10194-022-01418-8

**Published:** 2022-04-18

**Authors:** Rami Apelian, Lee Boyle, Joe Hirman, Divya Asher

**Affiliations:** 1 Huntington Headache and Neurology, 289 W. Huntington Drive, Suite 301, Arcadia, CA 91007 USA; 2 Lundbeck LLC, Deerfield, IL USA; 3 Pacific Northwest Statistical Consulting, Inc., Woodinville, WA USA

**Keywords:** Eptinezumab, Dose response, Patient subgroups, Migraine

## Abstract

**Background:**

Eptinezumab 100 mg and 300 mg met the primary efficacy endpoint in both PROMISE clinical trials, significantly reducing frequency of monthly migraine days over Weeks 1‒12. The objective of this analysis was to assess the clinical response to eptinezumab 100 mg and 300 mg within the pivotal phase 3 PROMISE-1 and PROMISE-2 studies to potentially identify subsets of patients with meaningful differences between doses.

**Methods:**

Patients from PROMISE-1 (NCT02559895) and PROMISE-2 (NCT02974153) trials were divided into subgroups based on demographic and migraine characteristics, and baseline questionnaire responses. For each subgroup, the overall likelihood of achieving ≥ 50% migraine responder rate (MRR) over Weeks 1–12 and Weeks 13–24 with either eptinezumab 100 mg or 300 mg was calculated using odds ratios (with associated confidence intervals) and compared.

**Results:**

In PROMISE-1 (episodic migraine) and PROMISE-2 (chronic migraine), the likelihood of achieving ≥ 50% MRR over Weeks 1–12 and Weeks 13–24 was roughly equivalent for patients receiving either dose level of eptinezumab. Given the number of comparisons performed, sporadic apparent differences were seen but no replicated patterns between studies emerged. In PROMISE-1, no differences were observed in any subgroup over Weeks 1–12. In PROMISE-2, patients reporting < 15 monthly migraine days at baseline, *any problems* with mobility per the EQ-5D-5L, or a social functioning score > 45.0 per the 36-item Short-Form Health Survey (SF-36), appeared more likely to achieve ≥ 50% MRR with 300 mg over Weeks 1–12, with none of these being apparent in PROMISE-1.

**Conclusions:**

Overall, these data suggest that across PROMISE-1 and PROMISE-2, there were no meaningful differences in the likelihood of achieving ≥ 50% MRR between the eptinezumab dose levels in the majority of patient subgroups. In the few subgroups that displayed small, but potentially meaningful differences, patients were more likely to achieve ≥ 50% MRR with eptinezumab 300 mg; however, minimal consistency across both studies and time periods was noted.

**Trial Registration:**

ClinicalTrials.gov.

PROMISE-1: NCT02559895.

PROMISE-2: NCT02974153.

## Background

Migraine is a highly prevalent neurological disease resulting in a large burden on society and individuals. It is estimated that over 1 billion individuals have migraine [1, 2]. Women are more likely to have migraine than men, and migraine reduces quality of life, particularly during young adulthood and middle age [1].

Calcitonin gene-related peptide (CGRP) plays a major role in the pathophysiology of migraine and is a key target for many preventive migraine therapies [3]. Eptinezumab is a humanized IgG1 monoclonal antibody administered intravenously (IV) that binds CGRP with high affinity and specificity [4]. Eptinezumab at 100 mg and 300 mg achieves 90% of the maximal efficacy (EC_90_) after a single dose [5], suggesting that after initial IV infusion with eptinezumab 100 mg, most patients have sufficient levels of eptinezumab to sequester CGRP and prevent CGRP from interacting with its receptor. Eptinezumab was approved by the US Food and Drug Administration (FDA) in February 2020 for the preventive treatment of migraine [6]. In pivotal phase 3 studies (PROMISE-1 and PROMISE 2), the primary efficacy endpoint, i.e., reduction in mean monthly migraine days (MMDs) over Weeks 1‒12 and a prespecified secondary endpoint, i.e., patients experiencing a ≥ 50% reduction in MMDs, when administered as a preventive therapy, was met by the eptinezumab dose levels of 100 mg and 300 mg in both trials [7, 8]. Per the FDA-approved prescribing label, eptinezumab 100 mg is the recommended dose, although 300 mg may be beneficial for some patients [6].

When considering individualized and preventive migraine treatment, it is important to evaluate available data for evidence regarding whether patients with specific clinical attributes respond preferentially to a particular dose level of eptinezumab, which may inform optimal starting dose level and decisions for dose adjustments. While these analyses show us general trends in the average results of select patient subgroups, it is often challenging to adapt these data to predict individual patient responses. These analyses are limited by the inclusion/exclusion criteria of the trials, which differ from the real-world evaluation of patients. However, it is important to understand available data to make informed clinical decisions.

These subgroups of patients are based upon different demographics (e.g., age, sex), with varying levels of disease severity, or with concurrent conditions (e.g., obesity, medication-overuse headache [MOH]), who may respond preferentially to a specific dose. The objective of this post hoc analysis was to compare data from the various scales and assessments used in both PROMISE trials to determine if there is a difference in response across multiple clinical domains between the doses in any subset of patients.

## Methods

### Studies

PROMISE-1 (NCT02559895) and PROMISE-2 (NCT02974153) were double-blind, randomized, placebo-controlled, parallel-group studies. Study protocols have been published previously [7, 8]. For both clinical trials, the primary endpoint was change in frequency of migraine days over Weeks 1‒12. The ≥ 50% migraine responder rate (MRR), captured over Weeks 1‒12 and 13‒24, was a prespecified secondary endpoint, where patients achieving ≥ 50% MRR are those with a reduction of ≥ 50% in number of MMDs. The ≥ 50% MRR was selected as the endpoint for comparison as it is commonly used to evaluate preventive migraine treatment and is a metric that is comparable across the studies evaluated here. It also normalizes the variation inherent in MMDs (i.e., change from baseline). In addition, the 50% MRR coincides with the International Headache Society guidelines and the American Headache Society Position Statement on setting realistic expectations with use of advanced preventive therapy [9, 10].

PROMISE-1 included patients with episodic migraine (EM) and randomized eligible patients to receive eptinezumab 30 mg, 100 mg, 300 mg, or placebo, administered intravenously on Day 0 and every 12 weeks through Week 36 (i.e., up to 4 doses) [8]. Post-dose clinic visits occurred at Weeks 4, 8, 12, 16, 20, 24, 28, 36, 48, and 56. In PROMISE-1, MOH diagnosis and body mass index (BMI) were not used to determine inclusion/exclusion from the study. Baseline MMDs across all treatment groups were ~ 8.6, and baseline MHDs across all treatment groups were ~ 10.1.

PROMISE-2 included patients with chronic migraine (CM) and randomized eligible patients to receive eptinezumab 100 mg and 300 mg, or placebo, administered intravenously on Day 0 and Week 12 (i.e., up to 2 doses) [7]. Post-dose clinic visits occurred at Weeks 2, 4, 8, 12, 16, 20, 24, and 32. Patients with MOH not associated with opioid analgesics or barbiturate compounds were eligible for the study, and 40.5% (286/706) of eptinezumab-treated patients had MOH diagnosis at baseline (100 mg, *n* = 139; 300 mg, *n* = 147). Patients with a BMI ≥ 39 kg/m^2^ at screening were excluded from the study. Baseline MMDs across all treatment groups were ~ 16.1, and baseline MHDs across all treatment groups were ~ 20.4.

### Patient subgroups

Patients were divided into subgroups based on patient characteristics, baseline migraine characteristics, and baseline patient-reported outcome responses. Patient characteristics included BMI (i.e., normal/underweight, overweight, obese), age (i.e., > 40 and ≤ 40 years), and MOH diagnosis (i.e., yes or no; PROMISE-2 only). Migraine characteristics consisted of baseline MMDs, baseline MHDs, history of migraine with aura (i.e., yes or no), average headache length (hours), and patient-identified most bothersome symptom (PI-MBS; PROMISE-2 only). Validated patient questionnaires consisted of the EuroQol 5-dimension, 5-level scale (EQ-5D-5L), 36-item Short-Form Health Survey (SF-36; v2.0), and 6-item Headache Impact Test (HIT-6; PROMISE-2 only).

BMI subgroups are in alignment with weight status categories indicated by the US Food and Drug Administration [11], and age subgroups are in alignment with the median age of the US population (38.3 years in 2019) [12]. Thresholds defining MMD and MHD subgroups in the PROMISE-1 study have been used to distinguish between low-frequency and high-frequency episodic migraine [13–20]. Eligibility for PROMISE-2 included ≥ 15 MHDs, including ≥ 8 MMDs; thus, the cutpoints used for those subgroup definitions represented 1 week above those values.

Groupings for the PI-MBS were based on those established previously for validation [21, 22]. “Pain related to migraine” included eye pain, headache, pain, pain—anatomical, pain with activity, and throbbing/pulsation; “cardinal/traditional MBS” included nausea/vomiting, sensitivity to light, and sensitivity to sound; and “other symptoms” included allodynia, aura, cognitive disruption, dizziness, fatigue, inactivity, mood changes, neck pain, pressure/tightness, sensitivity to smell, sensory disturbance, sleep disturbance, speech difficulty, vision impacts, multiple, and other.

The EQ-5D-5L consists of five dimensions of health and the patient’s self-rated health [23]. Dimensions include mobility, self-care, usual activities, pain/discomfort, and anxiety/depression. For each dimension, the patient must select one of five responses: no problems, slight problems, moderate problems, severe problems, and extreme problems. Self-rated health is recorded on a visual analogue scale from “the best health you can imagine” (100) to “the worst health you can imagine” (0). For creating subgroups, responses on all dimensions were divided into two distinct groups: responses of no problems and responses of slight, moderate, severe, or extreme problems, representing those with no issues versus those with any issues in the respective domains. For the self-rated health scale, scores were divided into two response categories, > 80 or ≤ 80, based on the normative US population mean [24].

The SF-36 (v2.0) comprises 36 questions that measure functional health and well-being across eight domains [25]. These domains include physical functioning (10 items), role-physical (4 items), bodily pain (2 items), general health (5 items), vitality (4 items), social functioning (2 items), role-emotional (3 items), and mental health (5 items). These individual domains are then able to be combined to calculate a physical component summary score and a mental component summary score. The SF-36 utilizes a normative-based scoring system, which is the standardization of all SF-36 domains and component summary scores to a scale from 0 to 100 with mean of 50 and a standard deviation of 10, designed to be representative of the general US population [25]. To create subgroups, baseline scores were dichotomized into subgroups representing a more favorable health state (> 45.0) versus a less favorable health state (≤ 45.0), representing half a standard deviation below the normative US population mean of 50.

The HIT-6 (PROMISE-2 only) is a questionnaire used to measure the impact on the ability to function normally in daily life when a headache occurs [26]. It is a 6-question, Likert-type, self-reporting questionnaire (response scores: never = 6, rarely = 8, sometimes = 10, very often = 11, and always = 13), which assessed: 1) “When you have headaches, how often is the pain severe?”; 2) “How often do headaches limit your ability to do usual daily activities including household work, work, school, or social activities?”; 3) “When you have a headache, how often do you wish you could lie down?”; 4) “In the past 4 weeks, how often have you felt too tired to do work or daily activities because of your headaches?”; 5) “In the past 4 weeks, how often have you felt fed up or irritated because of your headaches?”; and 6) “ In the past 4 weeks, how often did your headaches limit your ability to concentrate on work or daily activities?” The total score for the HIT-6 is calculated from summing individual items (score range of 36‒78 points), with score ranges representing severe impact =  ≥ 60, substantial impact = 56–59, some impact = 50–55, and little to no impact =  ≤ 49. HIT-6 total score subgroups were based on the threshold for severe life impact at baseline, of which most patients were severe (eptinezumab 100 mg, 89.6%; eptinezumab 300 mg, 88.6%; and placebo, 87.4%). For each HIT-6 item, patients were divided into two distinct subgroups: those who responded never, rarely, or sometimes (i.e., patients who were less severely impacted) and those who responded very often or always (i.e., patients who were more severely impacted).

### Statistical analysis

Eptinezumab 100 mg and 300 mg were compared by calculating the odds ratio of achieving ≥ 50% MRR over Weeks 1‒12 and over Weeks 13‒24 in PROMISE-1 and in PROMISE-2.

The odds ratios and 95% confidence intervals (CIs) were calculated from logistic regression; p-values were not calculated because of the post hoc nature of this analysis. Each subgroup was run through a separate model with the ≥ 50% migraine responder status as the response variable and treatment (100 mg vs 300 mg) as the predictor variable. If the 95% CI crossed 1, it was interpreted that there was no meaningful difference in efficacy between the two doses of eptinezumab.

No attempt has been made to control for multiplicity; however, this analysis has been designed to allow for a degree of independent replication. The results from the two studies have been analyzed separately and for the two dosing intervals (Weeks 1‒12 and 13‒24) with the goal of identifying factors observed in both studies versus those seen in only one study. This independent replication may allow for an opportunity to identify random results, which are expected to occur when many repeat comparisons are performed, versus real and repeatedly observed effects.

In evaluating the results presented in this manuscript, it is important to understand the statistical properties of the analyses performed. The goal of these analyses is not to posit that 100 mg and 300 mg are identical; there is no clinical or biologic reason to assume exact equivalence of these doses. The goal is instead to determine if meaningful differences might exist. The definition of meaningful is, by necessity, left to the reader. As a data reduction step, this manuscript focuses on cases when the CI of the odds ratio fails to cover 1. However, from the point of view of false-positive and false-negative findings, we know that situations where the CI does not cover 1 may be false positive and that cases where the CI does cover 1 do not indicate that the two treatments are identical. Given that 95% CIs are being used, we expect that 1 in 20 CIs will fail to cover 1 even if the “truth” was exact equivalence. We also know that the sample size used for the various CIs limits what size of effects might be called out using this 95% interval data reduction approach. To attempt to address these issues, results have been presented for both studies individually and for two separate time points to mimic the concept of experimental replication within the confines of the available data.

## Results

Baseline demographics and characteristics from PROMISE-1 and PROMISE-2 have been previously reported [7, 8].

### Odds ratios of ≥ 50% migraine response over weeks 1–12

**Fig. 1:**
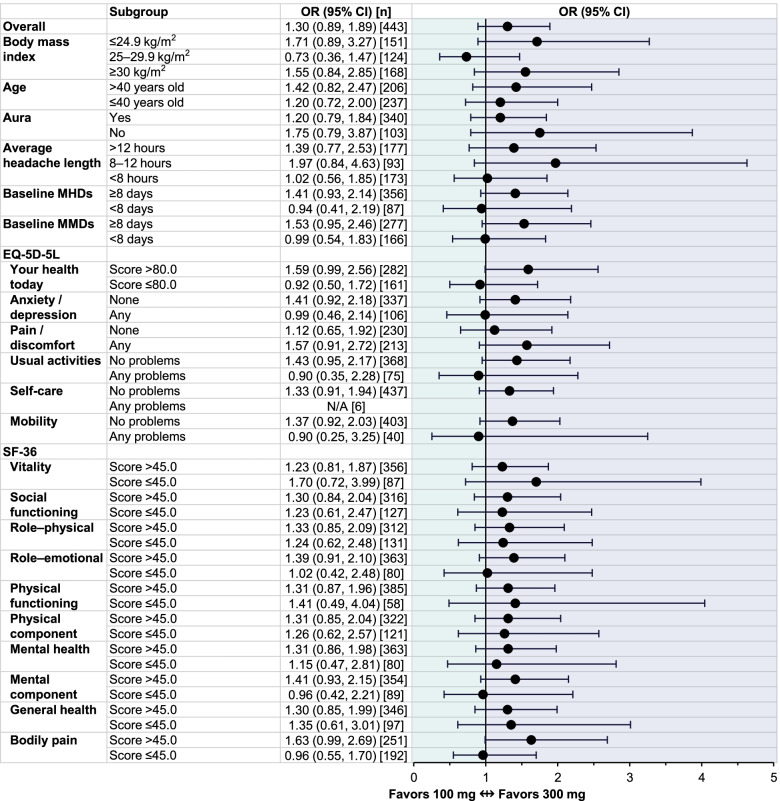
Odds ratio of ≥ 50% MRR over Weeks 1‒12 in PROMISE-1 subgroups defined by baseline demographics and patient-reported outcome measures. CI, confidence interval; EQ-5D-5L, EuroQol 5-dimension, 5-level scale; MHDs, monthly headache days; MMDs, monthly migraine days; MRR, migraine responder rate; N/A, not applicable (sample size too small); OR, odds ratio; SF-36, 36-item Short-Form Health Survey

In PROMISE-1 (comprising patients with EM), the overall likelihood of achieving ≥ 50% MRR over Weeks 1‒12 was approximately equivalent for patients receiving either eptinezumab 100 mg or 300 mg, with a slight, but not statistically meaningful, numerical advantage for the 300-mg dose (Fig. 1). There was no statistically meaningful difference in eptinezumab 100-mg and 300-mg efficacy across any of the subgroups, including those defined by BMI (≤ 24.9, 25–29.9, or ≥ 30 kg/m^2^), age (> 40 or ≤ 40 years), history of migraine with aura (yes or no), or average length of headache (> 12, 8–12, or < 8 h), baseline MHD frequency (≥ 8 or < 8 days), and baseline MMD frequency (≥ 8 or < 8 days), with all 95% CIs of the estimated odds ratios crossing the 1 threshold. In subgroups defined by EQ-5D-5L and SF-36 scores at baseline, the 95% CIs crossed the 1 threshold, indicating no statistically meaningful distinction in efficacy based on health-related quality of life between the two eptinezumab dose levels. For the EQ-5D-5L subgroup reporting any problems in response to the questions covering self-care, the sample size was too small to generate an accurate odds ratio.

**Fig. 2:**
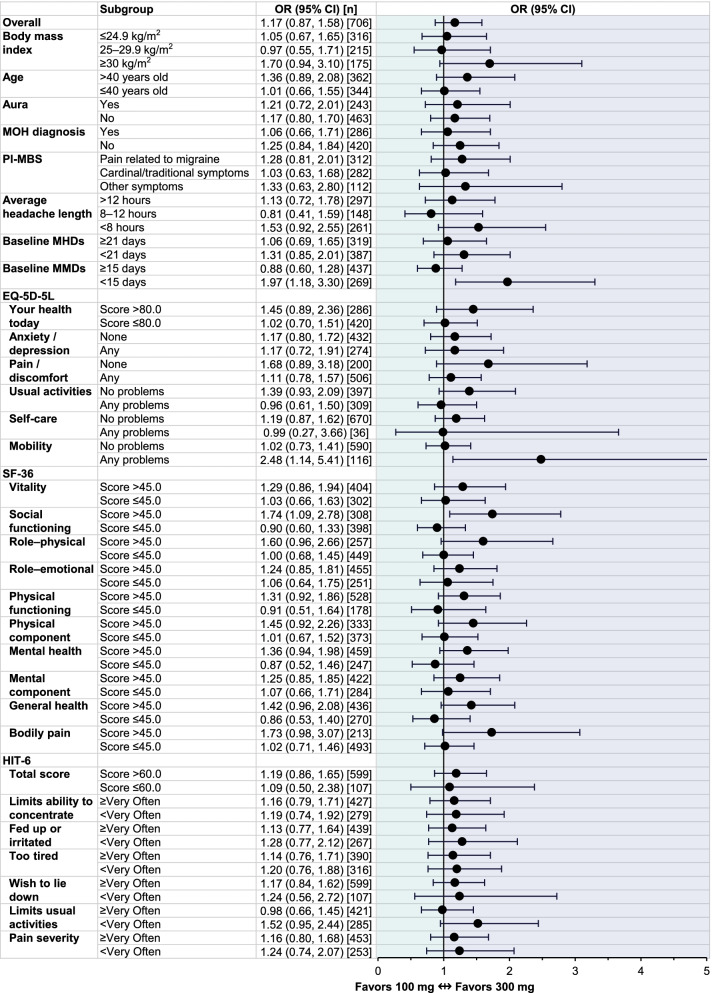
Odds ratio of ≥ 50% MRR over Weeks 1‒12 in PROMISE-2 subgroups defined by baseline demographics and patient-reported outcome measures. CI, confidence interval; EQ-5D-5L, EuroQol 5-dimension, 5-level scale; HIT-6, 6-item Headache Impact Test; MHDs, monthly headache days; MMDs, monthly migraine days; MRR, migraine responder rate; OR, odds ratio; PI-MBS, patient-identified most bothersome symptom; SF-36, 36-item Short-Form Health Survey

In PROMISE-2 (comprising patients with CM), the overall likelihood of achieving ≥ 50% MRR over Weeks 1‒12 was roughly equivalent for patients receiving either dose (eptinezumab 100 mg or 300 mg) (Fig. 2). Regardless of BMI, age, history of migraine with aura, MOH diagnosis (yes or no), PI-MBS category, average length of headache, and baseline MHD frequency (≥ 21 or < 21 days), there did not appear to be a statistically meaningful difference in efficacy between the two eptinezumab doses according to the odds ratio and 95% CIs. For the subgroup with ≥ 15 MMDs at baseline, there was no statistically meaningful difference in efficacy; however, in the subgroup with < 15 MMDs at baseline, the odds ratio (1.97; 95% CI: 1.18, 3.30) indicated that eptinezumab 300 mg had slightly higher efficacy over 100 mg.

Most PROMISE-2 subgroups defined by EQ-5D-5L and SF-36 scores at baseline did not demonstrate a clear benefit of either dose. For patients with any mobility issues per the EQ-5D-5L and for patients with a score > 45.0 for the SF-36 social functioning domain (more favorable health state), the odds ratio of response (EQ-5D-5L mobility, 2.48 [95% CI: 1.14, 5.41]; SF-36 social functioning, 1.74 [95% CI: 1.09, 2.78]) suggested that those receiving eptinezumab 300 mg were more likely to achieve ≥ 50% MRR. For subgroups defined by HIT-6 responses, the results suggested no clear benefit of either dose of eptinezumab, as all 95% CIs of the estimated odds ratios crossed the 1 threshold.

### Odds ratios of ≥ 50% migraine response over weeks 13–24

**Fig. 3:**
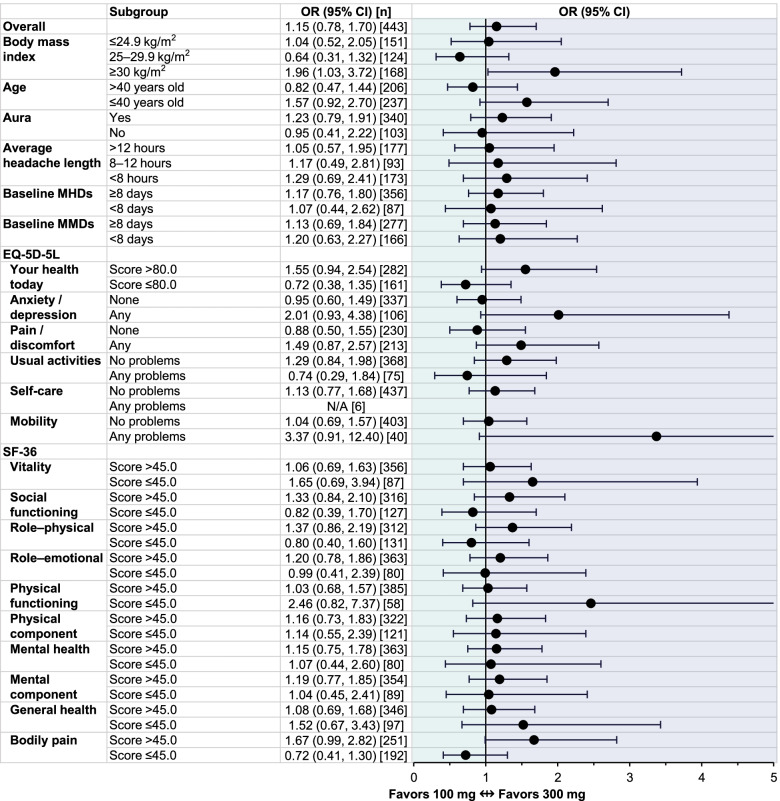
Odds ratio of ≥ 50% MRR over Weeks 13‒24 in PROMISE-1 subgroups defined by baseline demographics and patient-reported outcome measures. CI, confidence interval; EQ-5D-5L, EuroQol 5-dimension, 5-level scale; MHDs, monthly headache days; MMDs, monthly migraine days; MRR, migraine responder rate; N/A, not applicable (sample size too small); OR, odds ratio; SF-36, 36-item Short-Form Health Survey

For eptinezumab-treated patients with EM in the PROMISE-1 study, the overall likelihood of achieving ≥ 50% MRR over Weeks 13–24 (i.e., after a second infusion) was not meaningfully different for patients receiving either 100 mg or 300 mg (Fig. 3). Non-obese subgroups (BMI ≤ 29.9 kg/m^2^) did not appear to have different responses to one dose over the other; however, patients who were obese (BMI ≥ 30.0 kg/m^2^) were slightly more likely to achieve ≥ 50% MRR with eptinezumab 300 mg (odds ratio, 1.96 [1.03, 3.72]). There did not appear to be meaningful differences with either dose across subgroups defined by age, history of migraine with aura, average length of headache, baseline MHD frequency, baseline MMD frequency, or EQ-5D-5L or SF-36 scores.

**Fig. 4:**
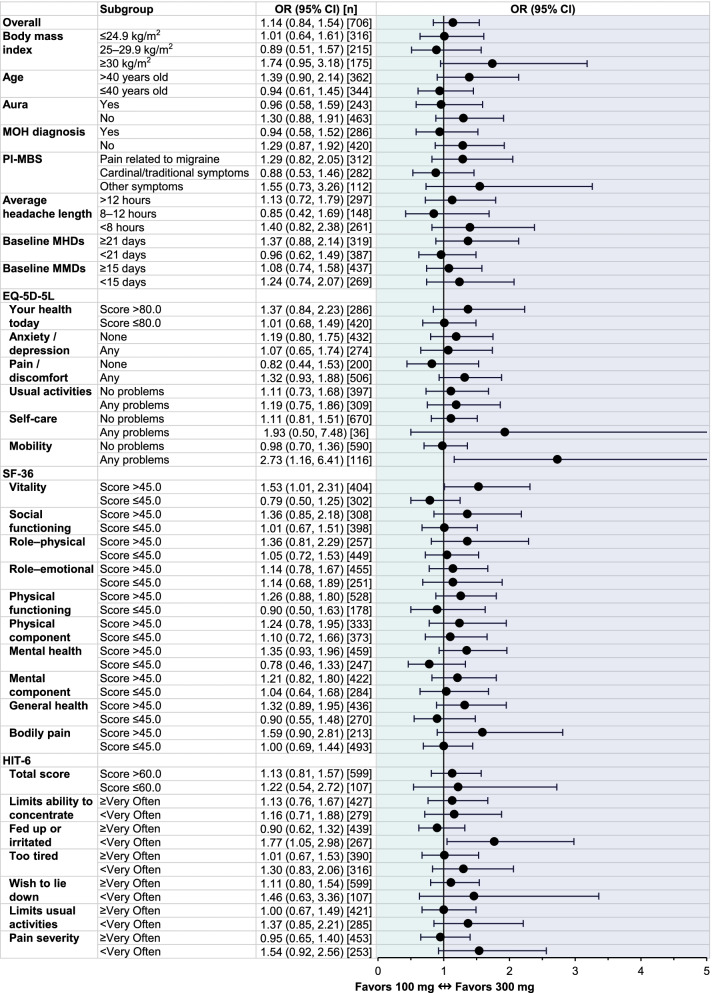
Odds ratio of ≥ 50% MRR over Weeks 13‒24 in PROMISE-2 subgroups defined by baseline demographics and patient-reported outcome measures. CI, confidence interval; EQ-5D-5L, EuroQol 5-dimension, 5-level scale; HIT-6, 6-item Headache Impact Test; MHDs, monthly headache days; MMDs, monthly migraine days; MRR, migraine responder rate; PI-MBS, patient-identified most bothersome symptom; OR, odds ratio; SF-36, 36-item Short-Form Health Survey

In PROMISE-2 (CM), the overall likelihood of achieving ≥ 50% MRR after receiving two doses of eptinezumab (administered on Day 0 and Week 12) was roughly equivalent for patients receiving either eptinezumab 100 mg or 300 mg (Fig. 4). Across patient subgroups defined by age, BMI, history of migraine with aura, MOH diagnosis, PI-MBS category, average length of headache, baseline MHD frequency, and baseline MMD frequency, the 95% CIs for the estimated odds ratios crossed 1, suggesting no meaningful difference in efficacy between the two doses of eptinezumab in any subgroup. In most EQ-5D-5L and SF-36 subgroups, the 95% CIs crossed 1, indicating no statistically meaningful difference in efficacy between the two eptinezumab doses. For patients who responded as having any problems with mobility (EQ-5D-5L; OR: 2.73 [1.16, 6.41]) or with a score > 45.0 for vitality (SF-36; OR: 1.53 [1.01, 2.31]), the odds ratio suggested those receiving eptinezumab 300 mg were more likely to have ≥ 50% MRR than those receiving eptinezumab 100 mg. For subgroups defined by HIT-6 responses, the results suggested no meaningful difference between the doses of eptinezumab in all subgroups, except for those who reported very often or always feeling “fed up or irritated” (1.77 [1.05 – 2.98]), who were patients more likely to achieve ≥ 50% MRR on 300 mg than 100 mg.

## Discussion

In patients with EM and CM, eptinezumab 100 mg and 300 mg both separately demonstrated statistically greater percentages of patients achieving ≥ 50% MRR compared with placebo [7, 8], as well as a favorable safety and tolerability profile across both doses [7, 8, 27, 28]. This post hoc analysis compared the odds ratios of achieving ≥ 50% MRR with eptinezumab 100 mg compared with 300 mg in patients from the phase 3 clinical trials with EM and CM to determine if there is a meaningfully preferential response to a specific dose in any subset of patients. Results of this analysis suggested that across both PROMISE-1 and PROMISE-2, there was no meaningful difference in ≥ 50% MRR between the doses across the majority of subgroups, including age, BMI, history of migraine with aura, average headache length, MOH diagnosis, baseline MMD and MHD frequencies, SF-36, EQ-5D-5L, and HIT-6 scores (PROMISE-2 only). Overall, the results across multiple patient demographics are likely to be correlated, but there is no clear indication of dosing preference given that these analyses did not explore whether patient characteristics were not seen consistently across studies or timepoints.

In the subgroups where the odds ratio was not considered statistically different (Table 1), the 300-mg dose had slightly higher efficacy; however, these results were not consistent between studies and time points. In patients with EM, the slightly higher differential response to 300 mg observed over Weeks 13–24 in the obese (BMI ≥ 30 kg/m^2^) subgroup was not observed during Weeks 1–12, nor was this observed for either dosing interval in PROMISE-2 (CM). Migraine patients are a heterogenous population [29], and this is supported by the demographics in our study population. Additionally, patients with migraine frequently transition between episodic and chronic status based on environmental exposures, exposure to triggers, response to preventive therapy, and response to abortive therapy [29]. While the data do not support that predefined characteristics can predict an early or late response to eptinezumab infusion at either the 100-mg or 300-mg dose, the data does demonstrate that the 300 mg dose across most demographic groups had numerically higher efficacy, though not statistically meaningful. Overall, these results highlight that individual patients’ needs must be factored into the clinician’s decision making and that each patient is unique.

**Table 1:** List of parameters measured in PROMISE-1 and PROMISE-2 where 300-mg was favored over 100-mg dose in predicting ≥ 50% migraine responder rate^a^

Time Point (Study)	Subgroup (dose favored)
Weeks 13‒24 (PROMISE-1)	Body mass index ≥ 30.0 kg/m^2^ (300 mg)
Weeks 1‒12 (PROMISE-2)	Baseline monthly migraine days < 15 (300 mg)
Weeks 1‒12 (PROMISE-2)	EQ-5D-5L any problems with mobility (300 mg)
Weeks 1‒12 (PROMISE-2)	SF-36 social functioning > 45.0 (300 mg)
Weeks 13‒24 (PROMISE-2)	EQ-5D-5L any problems with mobility (300 mg)
Weeks 13‒24 (PROMISE-2)	SF-36 vitality > 45.0 (300 mg)
Weeks 13‒24 (PROMISE-2)	HIT-6 fed up or irritated < very often (300 mg)

^a^Efficacy between 100-mg vs 300-mg doses was considered statistically different if the 95% confidence intervals for calculated odds ratio did not cross “1”

*EQ-5D-5L* EuroQol 5-dimension, 5-level scale, *HIT-6* 6-item Headache Impact Test, *SF-36* 36-item Short-Form Health Survey

In patients with CM, the higher likelihood of ≥ 50% MRR with 300 mg than with 100 mg over Weeks 1–12 was noted for patients with < 15 MMDs at baseline and the subgroup with an SF-36 social functioning score of > 45.0 at baseline; however, these differences were not seen over Weeks 13–24. Due to the heterogeneity in the migraine population which may not be adequately addressed in pooled population studies, it is important that the prescribing clinician consider all potential triggers or modifiable risk factors in every patient when deciding on therapeutic options.

Patients with CM who reported a more favorable vitality score per the SF-36 (> 45.0) or feeling fed up or irritated *never, rarely,* or *sometimes* per the HIT-6 showed a difference over Weeks 13–24 but not Weeks 1–12. Notably, the odds ratio of ≥ 50% MRR favored eptinezumab 300 mg across both time points in patients with CM who reported any problems with EQ-5D-5L mobility at baseline; that this was not observed in patients with EM may potentially reflect the increased likelihood of experiencing a migraine on the day of assessment in patients with CM than with EM. Additionally, it is important to recognize that HIT-6 is a migraine-specific tool, while the SF-36 is a general health questionnaire used to determine the measure of disability regardless of disease state. The HIT-6 data serves as a potent measure of patient outcomes in the migraine space and should be factored into the clinical decision algorithm in patients with CM, regardless of their vitality level on the SF-36.

Overall, the lack of clear differentiation between outcomes with eptinezumab 100 mg and 300 mg is in alignment with a recently published analysis that found the risk ratio of ≥ 50% MRR over Weeks 1–12 was 0.93 (95% CI: 0.85, 1.02; *P* = 0.11) [27]. The analysis also found no significant difference between doses for change from baseline in MMDs, percent of patients with a migraine on the day after first infusion, and rate of treatment-emergent adverse events; however, it did find that 300 mg was associated with higher ≥ 75% MRR over Weeks 1–12 (*P* = 0.01). The current analysis focuses on the ≥ 50% MRR as the outcome measure and evaluates the odds ratio of achieving that outcome over two separate dosing intervals and in multiple subgroups, finding no clear trend in whether a specific subgroup would benefit from a specific dose at either time point. Additionally, this analysis aimed to examine subgroups for potential differences rather than assessing how the doses affect patients across the migraine spectrum. Although the current analysis did not demonstrate any statistical outliers, individual patients may still require higher or lower doses based on efficacy and tolerability. The favorable side effect profile between the two doses allows the clinician flexibility in tailoring a treatment plan unique to each patient.

### Limitations

Due to the post hoc nature of this analysis, additional experimental studies are needed to confirm these findings. In addition, PROMISE-1 and PROMISE-2 were not explicitly designed to rigorously differentiate between active doses of eptinezumab. Further, the small sample size of some of the subgroups limited the statistical power for detecting differences between eptinezumab doses, and subgroups such as sex, race, and ethnicity were not fully representative of a real-world migraine population and thus were not included (i.e., study populations were 84–88% female, 84–91% white, and 82–92% non-Hispanic) [7, 8]. Additional subgroups defined by socioeconomic factors (e.g., education, employment, household income, and health coverage), migraine history (e.g., previous preventive medication, acute medication), and associated comorbidities (e.g., mood, and respiratory and cardiovascular disorders), were not consistently captured across PROMISE-1 and PROMISE-2 or were explicitly excluded from participation, thus preventing further analyses. Specifically, in PROMISE-1, patients had limited use of acute migraine medications (≤ 14 days per 28-day period) and of triptans (≤ 10 days per 28-day period) in the 3 months prior to screening and the 28-day period prior to randomization [8], and were required to use limited prophylactic headache medication or menstrual migraine prophylactics (≤ 7 days per 28-day period) within the 2 months prior to screening and during the 28-day period before randomization. Further, patients with cardiovascular or neurological disease, diabetes, life-threatening allergies, or high blood pressure at treatment were excluded. In PROMISE-2, patients with a BMI ≥ 39 kg/m^2^, uncontrolled or newly diagnosed hypertension, or known history or evidence of cardiovascular, neurological, or autoimmune disorders were excluded, and patients were required to have limited use of barbiturates or prescription opiates (≤ 4 days per month) [7]. This study also only evaluated the ≥ 50% MRR endpoint across Weeks 1‒12; however, the percentage of ≥ 75% migraine responders across Weeks 1‒12 were also similar between doses (PROMISE-1: 22.2%, 100 mg (*n* = 221); 29.7%, 300 mg (*n* = 222); PROMISE-2: 26.7%, 100 mg (*n* = 356); 33.1%, 300 mg (*n* = 350)) [6]. Additionally, patients transitioning into and out of ≥ 50% MRR between dosing intervals were not evaluated in this analysis, nor was the impact of Weeks 1–12 outcomes on Weeks 13–24 outcomes. While migraine response to eptinezumab is generally consistent across the course of treatment [30], some patients may change from being responders to non-responders and vice versa, as would be expected in real life; however, in this analysis, the conclusion is that the data do not support individual social or demographic characteristics influencing clinical decision making on dosing.

An additional limitation to this work was the lack of dose-crossover design; in these studies, patients were kept on the same treatment (eptinezumab 100 mg, 300 mg, or placebo) throughout the duration of the study. With the many subgroups included in this post hoc analysis, results between individual subgroups may be intercorrelated and therefore redundant, rather than additive, with interpretation limited by the lack of multivariant analysis to better inform patient-centered preventive migraine treatment. Hence, individual items where the confidence interval does not cover 1 may represent different aspects of the same underlying clinical condition. In addition, the statistical approach used to assess dose differences (confidence intervals surrounding odds ratios) may result in false positive and false negative findings; i.e., we cannot posit that cases where confidence intervals cross 1 definitively indicate that the doses have identical effects or vice versa. Future analyses evaluating individual patient-level response factors over time are necessary to determine the clinical relevance of these findings for an individual patient.

## Conclusions

Analyzing patient response to different eptinezumab doses across a multitude of patient subgroups demonstrated that for most patient subgroups there was no meaningful difference in efficacy across doses; however, a lack of meaningful difference between subgroups does not indicate there will be no difference between doses for any individual patient. In certain subgroups, eptinezumab 300 mg showed a slight numerical advantage compared with eptinezumab 100 mg in achieving ≥ 50 MRR over Weeks 1–12 or 13–24. Overall, these results support individualized preventive migraine treatment and should particularly inform patient discussions regarding appropriate eptinezumab doses.

## Data Availability

Data Sharing Statement: In accordance with EFPIA’s and PhRMA’s “Principles for Responsible Clinical Trial Data Sharing” guidelines, Lundbeck is committed to responsible sharing of clinical trial data in a manner that is consistent with safeguarding the privacy of patients, respecting the integrity of national regulatory systems, and protecting the intellectual property of the sponsor. The protection of intellectual property ensures continued research and innovation in the pharmaceutical industry. Deidentified data are available to those whose request has been reviewed and approved through an application submitted to https://www.lundbeck.com/global/our-science/clinical-data-sharing.

## Abbreviations

BMI: Body mass index
CGRP: Calcitonin gene-related peptide
CI: Confidence interval
CM: Chronic migraine
EM: Episodic migraine
EQ-5D-5L: EuroQol 5-Dimension, 5-Level scale
FDA: Food and Drug Administration
HIT-6: 6-Item Headache Impact Test
IV: Intravenous
MHDs: Monthly headache days
MMDs: Monthly migraine days
MOH: Medication-overuse headache
MRR: Migraine responder rate
PI-MBS: Patient-identified most bothersome symptom
SF-36: 36-Item Short-Form Health Survey
